# Optimal heat emissivity control of n population system based on individual size-distribution

**DOI:** 10.1038/s41598-023-42648-3

**Published:** 2023-10-18

**Authors:** Zhenggang Ba, Ye Wang

**Affiliations:** 1 School of Environmental and Municipal Engineering, Lanzhou Jiaotong University, Lanzhou, 730070 China; 2 Key Laboratory of Railway Vehicle Thermal Engineering, Ministry of Education of China, Lanzhou, 730070 China

**Keywords:** Ecology, Mathematics and computing

## Abstract

This paper generalizes a class of controllability problems based on the scale structure population system model. Based on the comparison principle of linear systems, the solution of the nonlinear system model is obtained by referring to the fixed point theorem. The non-negative, boundedness, existence, and uniqueness of the solution of the system model are established. The optimality condition is described in detail by means of a normal cone and conjugate system under the condition of proving the continuous dependence of the state environment on the solution to control variables.

## Introduction

A study on the optimal control of research population system models. Many scholars agree on two points on this issue: one is to maintain the diversity of biological populations, saving endangered species, making the population evolve in a direction that is beneficial to human health, the second is to discover valuable and challenging mathematical control problems, expanding the boundaries of multi-swarm system control theory. At present, the research on this aspect has formed the conclusion, that research results refine people's perceptions and are recognized by people.

A large number of research literature show that the individual scale structure determines the growth and development factors of biological populations, it can more objectively and accurately verify the dynamic properties of the individual structure model of the population than the age structure. Thus, the problem of the population system model with scale structure has been widely concerned by many scholars. The main research results are as follows: references^1–4^ elaborated on the establishment process of this kind of model in detail, and deduced the maximum value principle by using the linear comparison. References^5–7^ studied the local existence of the solution of the scale-structured principle, population model and its dependence on the initial conditions, the local existence and global boundedness of the positive solution of the model. Reference^8^ established and discussed the population model of scale structure and its optimal controllability, proved the existence principle of understanding, and finally obtained the effectiveness and feasibility of the optimal harvesting strategy. Reference^9^ extended the two-population model to three-population system model on the basis of reference^10^. However, there are two slight deficiencies in the literature reported above: (1) The population mathematical models established in the aforementioned literature tend to be limited to three population system models. From the perspective of the actual natural ecosystem, a benign biological ecological food chain is characterized by the coexistence of multiple groups of individuals. (2) The aforementioned literature hardly gives detailed proof for the optimization strategy, neglecting the constraint relationship between the external environment and the individual scale. (3) Although the female ratio was introduced, But the optimal gestation period for females in the population system is almost ignored, this is against the law of biological reproduction. In view of this, this paper establishes a multi-swarm system model with scale distribution. The purpose is to introduce the control factors that the external environment has on the individual scale of the population, and the average reproductive rate of female individuals is taken into account. After deriving the non-negativity, boundedness, and uniqueness of the solution of the system model, the optimization strategy is proved in detail. It highlights that the radiation of the environment to individuals is not only related to time but also to scale, namely, emissivity is a binary function of scale and time. It makes the ecosystem model more consistent with reality and the individual optimization of the population system move towards a virtuous circle of development.

## Construction of population system model

1 $$\left\{ \begin{gathered} \frac{{\partial p_{i} }}{\partial t} + \frac{{\partial (g_{i} (s)p_{i} )}}{\partial s} = - \mu_{i} (s,t)p_{i} - \sum\limits_{k = 1,k \ne i}^{n} {\lambda_{ik} (s,t)P_{k} (t)p_{i} } + \upsilon_{i} (s,t), \hfill \\ g_{i} (0)p_{i} (0,t) = \beta_{i} (t)\int_{{s_{1} }}^{{s_{2} }} {m_{i} (s,t)p_{i} (s,t)ds,} \hfill \\ p_{i} (s,t) = p_{i0} (s), \hfill \\ P_{i} (t) = \int_{0}^{m} {p_{i} (s,t)ds,i = 1,2, \cdots ,n.} \hfill \\ \end{gathered} \right.$$

where the constant m represents the maximum scale value of the individual, the state variable ${p}_{i}(s,t)$ represents the density distribution function of the individual scale s of the i-th population at time t, $g_{i} (s) = \tfrac{{ds_{i} }}{dt}$ represents the scale relative growth rate of the i-th population individual, $\mu_{i} (s,t)$ is an additional death limit for the i-th individual due to intra-tribal competition, $\beta_{i} (t)$ is the average reproductive rate of females $m_{i} (s,t)$ is the proportion of female individuals, $\lambda_{i} (s,t)$ represents the interaction coefficient between individuals, T is the control period. $\upsilon_{i} (s,t)$ represents the individual-scale emissivity of the external environment to the i-th population, thus, it is the control variable. $\upsilon_{i} (s,t)$ represents the initial distribution of the radiance added by the external environment to the i-th population, thus, it is the control variable.

Allow control set: $V_{ad} = \left\{ {\left. {\upsilon_{i} \in L^{\infty } (Q):0 \le E_{i1} \le \upsilon_{i} (s,t) \le E_{i2} } \right\}} \right.$

2 $$E_{ij} \in L^{\infty } (Q)\quad j = 1,2.\quad i = 1,2, \cdots ,n.$$

The core content of this paper is to study the following optimal control:

3 $$\mathop {\min }\limits_{{\upsilon \in V_{ad} }} J(\upsilon )\underline{\underline{\Delta }} \sum\limits_{i = 1}^{n} {\int_{Q} {[\left| {p_{i}^{{\upsilon_{i} }} - e_{id} } \right|^{2} + \phi \left| {\upsilon_{i}^{*} } \right|^{2} ]} } dsdt.$$

Thus, $\upsilon = (\upsilon_{1} ,\upsilon_{2} , \cdots ,\upsilon_{n} ) \in V_{ad}$, $p^{\upsilon } = (p_{1}^{{v_{1} }} ,p_{2}^{{\upsilon_{2} }} , \cdots ,p_{n}^{{\upsilon_{n} }} )$ is the solution of system (1) corresponding to the $\upsilon = (\upsilon_{1} ,\upsilon_{2} , \cdots ,\upsilon_{n} )$ nonnegative bounded function $e_{d} (s,t) = (e_{1} ,e_{2} , \cdots ,e_{n} ) \in L^{\infty } (Q)$ represents the density distribution of the observed population in a near-ideal state after the individual receives the radiant heat energy from the external environment,$\phi$ is the absorption weighting coefficient of the individual after the external environment irradiates the individual, and $0 \le \phi < 1$, $Q = (0,S) \times (0,T)$, S is the largest finite scale of the individual. According to the needs of actual research, the following basic assumptions are given here:

$(A_{1} )$ For any $g_{i} (s) \in L^{1} [0,S]$, when $s \in \left[ {0,S} \right)$, there are $g_{i}^{\prime } (s) \le 0$ and ${\text{g}}_{{\text{i}}} (S) = 0$,$\tfrac{{g_{i} (0)}}{{g_{i} (s)}} \le 1$ holds almost everywhere on $(s,t) \in Q_{T}$.

$(A_{2} )$ $\mu_{i} ( \cdot ,t) \in L_{loc(0,m)}^{1} ,\mu_{i} ( \cdot ,t) \ge 0$, holds almost everywhere on $(s,t) \in Q_{T}$.

$(A_{3} )$ $\beta_{i} (s,t) \in L^{\infty} (Q_{T}), \beta_{i} (s,t) \ge 0$, $\beta_{i} (s,t) \ge 0$ holds almost everywhere on $(s,t) \in Q_{T}$.

$(A_{4} )$ $p_{i0} (s) \in l^{\infty } (0,S_{ + } )$, $0 \le p_{i0} (s) \le N_{0}$, $N_{0} > 0$ is given constant, $\forall s \in (0,S)$.

## The uniqueness of the zero-point existence of the basic food chain model function

### Definition 1

Vector function $p = (p_{1} (s,t),p_{2} (s,t), \cdots ,p_{n} (s,t))$ is the solution of system (1), and satisfies the following integral Eq. (4), whose detailed descriptions can be found in relative Reference^11^:

4 $$\left\{ \begin{gathered} \int_{0}^{s} {p_{i} (a,t)da - \int_{0}^{s} {p_{i0} (a)da + \int_{0}^{t} {g_{i} (s)p_{i} (s,\theta )d\theta ,} } } \hfill \\ = \int_{0}^{t} {g_{i} (s)\beta_{i} (\theta )\int_{{s_{1} }}^{{s_{2} }} {m_{i} (s,\theta )p_{i} (s,\theta )dsd\theta - \int_{0}^{t} {\int_{0}^{s} {[\mu_{i} (a,\theta )p_{i} (a,\theta )} } } } \hfill \\ + \sum\limits_{k = 1,k \ne i}^{n} {\lambda_{ik} P_{k} (t)p_{i} (a,\theta ) - \upsilon_{i} (a,\theta )]dsd\theta ,} \hfill \\ P_{i} (\theta ) = \int_{0}^{m} {p_{i} (s,\theta )ds,i = 1,2, \cdots ,n.} \hfill \\ \end{gathered} \right.$$

### Theorem 1

If $(A_{1} )\sim (A_{2} )$ true, then for any given $\upsilon \in V_{ad}$, system (1) will be subject to the following.

Conditions:

$(B_{1} )$ System (1) has the only non-negative solution that is $p^{\upsilon }$.

$$(B_{2} )$$ $$0 \le p_{i}^{\upsilon } (s,t) \le p_{i} (s,t),\forall (s,t) \in Q,i = 1,2, \cdots ,n$$.

$(B_{3} )$ $p^{\upsilon }$ continuously depends on $\upsilon$.

Proof for $\forall k = (k_{1} ,k_{2} , \cdots ,k_{n} ) \in C(0,T;L^{2} (0,S)) \cap L^{\infty } (Q_{T} ;R^{n} )$, $Q_{T} = (0,S) \times (0,T)$, and $\upsilon_{i} \ge 0,$ the simulation function is $F_{i} = \int_{0}^{m} {f_{i} } (s,t)ds$, $(i = 1,2, \cdots ,n)$, refers to the solution of the following simulation system problem:

5 $$\left\{ \begin{gathered} \frac{{\partial p_{i} }}{\partial t} + \frac{{\partial (g_{i} (s)p_{i} )}}{\partial s} = - \mu_{i} (s,t)p_{i} - \sum\limits_{k = 1,k \ne i}^{n} {\lambda_{ik} (s,t)F_{k} (t)p_{i} } + \upsilon_{i} (s,t), \hfill \\ g_{i} (0)f_{i} (0,t) = \beta_{i} (t)\int_{{s_{1} }}^{{s_{2} }} {m_{i} (s,t)f_{i} (s,t)ds} . \hfill \\ \end{gathered} \right.$$

It is known from^4^ that the only non-negative solution of system (4) is $p^{f} = (p_{1}^{{f_{1} }} ,p_{2}^{{f_{2} }} , \cdots ,p_{n}^{{f_{n} }} ) \in L^{2} (Q_{T} ;R^{n} ),$ $p_{i}^{{f_{i} }} (s + ,t) = 0$,which by the principle of linear comparison to get $p_{i}^{f} \le \overline{{p_{i} }} ,$ and $\overline{{p_{i} }} \in L^{2} (Q_{T} ;R^{n} ) \cap L^{\infty } (Q_{T} ;R^{n} )$ is a bound solution of the following system (see, e.g.,^12^:

6 $$\left\{ \begin{gathered} \frac{{\partial y_{1} }}{\partial t} + \frac{{\partial (g_{1} (s)y_{1} )}}{\partial s} = - \mu_{1} (s,t)y_{1} + \upsilon_{1} (s,t), \hfill \\ g_{1} (0)y_{1} (0,t) = \beta_{1} (t)\int_{{s_{1} }}^{{s_{2} }} {m_{1} (s,t)y_{1} (s,t)ds,y_{1} (s,0) = p_{10} (s).} \hfill \\ \end{gathered} \right.$$

Generally speaking, when $f_{i - 1} (s,t) \le \overline{{p_{i - 1} }} (s,t)$, $p_{i}^{f} \le \overline{{p_{i} }} ,$ and $\overline{{p_{i} }} (i = 2, \cdots ,n)$ are a bounded solution for the following self-made system:

7 $$\left\{ \begin{gathered} \frac{{\partial y_{i} }}{\partial t} + \frac{{\partial (g_{i} (s)y_{i} )}}{\partial s} = - \mu_{i} (s,t)y_{i} + \upsilon_{i} (s,t), \hfill \\ g_{i} (0)y_{i} (0,t) = \beta_{i} (t)\int_{{s_{1} }}^{{s_{2} }} {m_{i} (s,t)y_{i} (s,t)ds,y_{i} (s,0) = p_{i0} (s).} \hfill \\ \end{gathered} \right.$$

For $$\forall f^{i} = (f^{1} ,f^{2} , \cdots ,f^{n} ) \in L^{2} (Q_{T} ;R^{n} ),$$$$0 \le f_{j}^{i} \le \overline{{p_{j} }}$$, we define the corresponding state variable of the self-made system as $$p^{i} = (p_{1}^{i} ,p_{2}^{i} , \cdots ,p_{n}^{i} ),$$ ($$i = 1,2, \cdots ,n$$), and define $$x = (x_{1} ,x_{2} , \cdots ,x_{n} ): = p^{1} - p^{2}$$, at this time, it can be obtained from system (6):

8 $$\left\{ \begin{gathered} \frac{{\partial x_{i} }}{\partial t} + \frac{{\partial (g_{i} (s)x_{i} )}}{\partial s} = - \mu_{i} (s,t)x_{i} - \sum\limits_{k = 1,k \ne i}^{n} {\lambda_{ik} (s,t)F_{k}^{i} (t)x_{i} } - \sum\limits_{k = 1,k \ne i}^{n} {\lambda_{ik} (s,t)(F_{k}^{1} - F_{k}^{2} (t))p_{i}^{2} } , \hfill \\ g_{i} (0)x_{i} (0,t) = \beta_{i} (t)\int_{{s_{1} }}^{{s_{2} }} {m_{i} (s,t)x_{i} (s,t)ds} , \hfill \\ x_{i} (m,0) = 0,F_{i}^{k} (t) = \int_{0}^{m} {f_{i}^{k} (s,t)ds,k = 1,2.i = 1,2, \cdots ,n.} \hfill \\ \end{gathered} \right.$$

Multiply both sides of the i-th (i = 1, 2,…, n) formula in system (7) by $x_{i}$, then integrating on *Q* can be obtained:

9 $$\left\| {x_{i} ( \cdot ,t)} \right\|_{{L^{2}_{(0,m)} }}^{2} \le c\sum\limits_{k = 1,k \ne i}^{n} {[\int_{0}^{t} {\left\| {f_{k}^{1} ( \cdot ,\tau ) - f_{k}^{2} ( \cdot ,\tau )} \right\|}_{{L_{(0,m)}^{2} }}^{2} d\tau } .$$

It's not hard to see that c is a constant that is not related to $f^{k} (k = 1,2)$, and $\left\| \cdot \right\|$ is the usual norm on space $L^{2} (0,S)$.

Assume closed set: $I: = f = (f_{1} ,f_{2} , \cdots ,f_{n} ) \in L^{2} (Q_{T} ;R^{2} );$ $0 \le f_{i} (s,t) \le \overline{{p_{i} }} ,$ $a.e.\forall (s,t) \in Q_{T} ,$

Define the mapping: $E:I \to I,$ $(Ef)(s,t): = p^{f} (s,t),\forall (s,t) \in Q_{T}$

The equivalent norm: $$\left\| f \right\|_{*} = \left\{ {\left\| {f_{1} } \right\|_{*}^{2} + \left\| {f_{2} } \right\|_{*}^{2} + \cdots + \left\| {f_{n} } \right\|_{*}^{2} } \right\}^{{\tfrac{1}{2}}} ,$$$$\left\| {f_{i} } \right\|_{ * } = \int_{0}^{T} {\left\| {f( \cdot ,t)} \right\|}^{2} \exp ( - 3ct)dt.$$

According to (9), it is easy to get:

$$\left\| {Ef^{1} - Ef^{2} } \right\|_{ * } = \left\| {P^{1} - P^{2} } \right\|_{ * } = \left\| {(x_{1} ,x_{2} , \cdots ,x_{n} )} \right\|_{ * } = \left\{ {\sum\limits_{i = 1}^{n} {\int_{0}^{T} {\left\| {x_{i} ( \cdot ,\tau )} \right\|^{2} } \exp ( - 3ct)dt} } \right\}^{{\tfrac{1}{2}}}$$

$$\begin{gathered} \le \left\{ {\sum\limits_{i = 1}^{n} {\int_{o}^{T} {\int_{0}^{t} {2c\left\| {f_{i}^{1} ( \cdot ,\tau ) - f_{i}^{2} ( \cdot ,\tau )} \right\|^{2} } } \exp ( - 3ct)d\tau dt} } \right\}^{{\tfrac{1}{2}}} \hfill \\ \le \left\{ {\frac{2}{3}\sum\limits_{i = 1}^{n} {\int_{0}^{T} {\left\| {f_{i}^{1} ( \cdot ,\tau ) - f_{i}^{2} ( \cdot ,\tau )} \right\|^{2} } \exp ( - 3c\tau )dt} } \right\}^{{\tfrac{1}{2}}} \hfill \\ = \frac{\sqrt 2 }{{\sqrt 3 }}\left\| {f^{1} - f^{2} } \right\|_{ * } \hfill \\ \end{gathered}$$

Thence, E is a compressed map on a complete space $(I,\left\| \cdot \right\|_{ * } )$, and there exists a unique fixed point that is a solution of system(1)on $\left[ {0,m} \right] \times [0,T]$.The following is a step-by-step analysis of the compact continuous dependence of this solution on $\upsilon .$

## Non-discontinuous dependence control of the solutions on the variable

Assume $\upsilon^{k} = (\upsilon_{1}^{k} ,\upsilon_{2}^{k} ,, \cdots \upsilon_{n}^{k} ) \in V_{ad} ,(k = 1,2.),$ $x_{i} = p_{i}^{{v^{1} }} - p_{i}^{{v^{2} }}$ $(i = 1,2, \cdots ,n.),$ it can be known from system (1) that $x_{i}$ is the solution of the following system:

10 $$\left\{ \begin{gathered} \frac{{\partial x_{i} }}{\partial t} + \frac{{\partial (g_{i} (s)x_{i} )}}{\partial s} = - \mu_{i} (s,t)x_{i} - \sum\limits_{k = 1,k \ne i}^{n} {\lambda_{ik} [x_{i} \int_{0}^{T} {p_{k}^{{v^{1} }} (t)ds + p_{i}^{{v^{2} }} \int_{0}^{m} {x_{k} ds} ]} } + \upsilon_{i}^{1} - \upsilon_{i}^{2} , \hfill \\ g_{i} (0)x_{i} (0,t) = \beta_{i} (t)\int_{{s_{1} }}^{{s_{2} }} {m_{i} (s,t)x_{i} (s,t)ds} ,x_{i} (m,0) = 0. \hfill \\ \end{gathered} \right.$$

Multiply both sides of the *i*th formula of the system (10) by $x_{i} (i = 1,2, \cdots ,n)$, and then integrate over *Q*, we can get that:

11 $$\left\| {x_{i} ( \cdot ,t)} \right\|_{{}}^{2} \le c_{i} [\sum\limits_{i = 1}^{n} {\int_{0}^{t} {\left\| {x_{i} ( \cdot ,\tau )} \right\|}_{{}}^{2} } d\tau + \int_{0}^{t} {\left\| {\upsilon_{i}^{1} ( \cdot ,\tau ) - \upsilon_{i}^{2} ( \cdot ,\tau )} \right\|}_{{}}^{2} d\tau ],i = 1,2, \cdots ,n.$$

where $c_{i}$ is a positive constant that does not depend on $\upsilon^{{\text{k}}}$ changes, and by (11), we get that:

12 $$\sum\limits_{i = 1}^{n} {\left\| {x_{i} ( \cdot ,\tau )} \right\|^{2} } \le \sum\limits_{i = 1}^{n} {[c_{i} \int_{0}^{t} {\left\| {x_{i} ( \cdot ,\tau )} \right\|}^{2} } d\tau + L\int_{0}^{t} {\left\| {\upsilon_{i}^{1} ( \cdot ,\tau ) - \upsilon_{i}^{2} ( \cdot ,\tau )} \right\|}^{2} d\tau ].$$

where ${\text{L:}} = {\text{max}} \{ {\text{c}}_{{1}}, {\text{c}}_{{2}}, \cdots,{\text{c}}_{{\text{n}}} \},$ and assumed ${\text{M}} = {\text{(c}}_{{1}} + {\text{c}}_{{2}} + \cdots + {\text{c}}_{{\text{n}}} {),}$ and L and M are both positive constants that do not depend on $\upsilon_{{\text{i}}}^{{\text{k}}} {\text{(k}} = {1,2)}$ combining (12), according to Gronwall's inequality, and integrating over (0, T), we get that:

13 $$\sum\limits_{i = 1}^{n} {\left\| {x_{i} ( \cdot ,\tau )} \right\|^{2} } \le LT(1 + MTe^{MT} )\sum\limits_{i = 1}^{n} {\left\| {\upsilon_{i}^{1} ( \cdot ,\tau ) - \upsilon_{i}^{2} ( \cdot ,\tau )} \right\|^{2} d\tau } .$$

To this end, from the above proof process, it can be determined that it $p^{\upsilon }$ is uniformly continuous with respect to $\upsilon$.

## Optimal analytical description of control elements

### Lemma 4.1

Let $\upsilon^{*} = (\upsilon_{1}^{*} ,\upsilon_{2}^{*} , \cdots ,\upsilon_{n}^{*} ),$ and $p^{*} = (p_{1}^{*} ,p_{2}^{*} \cdots ,p_{n}^{*} ),$ then $(\upsilon^{*} ,p^{*} )$ is the optimal pair of system (1) and control problem (2), while to any $\varepsilon > 0,$ and it sufficiently limited small, to any $\upsilon = (\upsilon_{1} ,\upsilon_{2} , \cdots ,\upsilon_{n} ) \in L^{2} (Q;R^{n} )$ and $\upsilon_{i} > 0,$ for any given $d = (d_{1} ,d_{2} , \cdots ,d_{n} ) \in L^{2} (Q;R^{n} )$, satisfy $\upsilon^{*} + \varepsilon d \in V_{ad}$, then the following limit is established:

$$\frac{1}{\varepsilon }(p^{{\upsilon^{ * } + ed}} - p^{{\upsilon^{ * } }} ) \to h\quad {\text{as}}\;\varepsilon \to 0^{ + } .$$

When $\upsilon^{*} = (\upsilon_{1}^{*} ,\upsilon_{2}^{*} , \cdots ,\upsilon_{n}^{*} )$ and $p^{*} = (p_{1}^{*} ,p_{2}^{*} \cdots ,p_{n}^{*} )$, then $h = (h_{1} ,h_{2} \cdots ,h_{n} )$ is an efficient control solution for the following self-made system (see, e.g.,^13–15^:

14 $$\left\{ \begin{gathered} \frac{{\partial h_{i} }}{\partial t} + \frac{{\partial (g_{i} (s)h_{i} )}}{\partial s} = - \mu_{i} (s,t)h_{i} - \sum\limits_{k = 1,k \ne i}^{n} {\lambda_{ik} (s,t)\left[ {P_{k}^{{\upsilon_{k}^{*} }} (t)h_{i} - H_{k} (t)p_{i}^{{\upsilon_{i}^{*} }} } \right]} + d_{i} (s,t), \hfill \\ g_{i} (0)h_{i} (0,t) = \beta_{i} (t)\int_{{s_{1} }}^{{s_{2} }} {m_{i} (s,t)h_{i} (s,t)ds} , \hfill \\ H_{i} (t) = \int_{0}^{m} {h_{i} (s,t)ds,h_{i} (m,0) = 0,} \hfill \\ P_{i}^{{\upsilon_{i}^{*} }} (t) = \int_{0}^{m} {p_{i}^{{\upsilon_{i}^{*} }} (s,t)ds,i = 1,2, \cdots ,n.} \hfill \\ \end{gathered} \right.$$

When $h(s,t)$ is the exact solution of the above heat transfer system, then $q = (q_{1} ,q_{2} , \cdots ,q_{n} )$ is the solution of the following self-made conjugate system (see^16–19^:

15 $$\left\{ \begin{gathered} \frac{{\partial q_{i} }}{\partial t} + g_{i} (s)\frac{{\partial q_{i} }}{\partial s} = - \mu_{i} (s,t)q_{i} + \sum\limits_{k = 1,k \ne i}^{n} {\lambda_{ik} } P_{k} (k)P_{k}^{{\upsilon_{k}^{*} }} q_{i} + (p_{i}^{{\upsilon_{i}^{*} }} - e_{id} ) \hfill \\ - \beta_{i} (s,t)m_{i} (s,t)g_{i} (0)q_{i} (0,t) + \sum\limits_{k = 2}^{n} {\lambda_{ik} } p_{k}^{{\upsilon_{k}^{*} }} \int_{0}^{m} {q_{k} (s,t)ds} , \hfill \\ q_{i} (s,T) = 0,P_{i}^{{\upsilon_{i}^{*} }} (t) = \int_{0}^{m} {p_{i}^{{\upsilon_{i}^{*} }} (s,t)ds,i = 1,2, \cdots ,n.} \hfill \\ \end{gathered} \right.$$

and then we must have:

16 $$\upsilon_{i}^{*} = \left\{ {\begin{array}{*{20}l} {E_{1} } \hfill & {E_{1} > \frac{{q_{i} (s,t)}}{\phi },} \hfill \\ {\frac{{q_{i} (s,t)}}{\phi }} \hfill & {E_{1} \le \frac{{q_{i} (s,t)}}{\phi } < E_{2} } \hfill \\ {E_{2} } \hfill & {E_{2} \le \frac{{q_{i} (s,t)}}{\phi }} \hfill \\ \end{array} } \right.$$

### *Proof*

To any $d = (d_{1} ,d_{2} \cdots d_{n} ) \in L^{\infty } (Q;R^{n} )$ and $(d_{i} \in T_{{\upsilon_{i} }} (\upsilon_{i}^{\varepsilon } )),T_{{\upsilon_{i} }} (\upsilon_{i}^{\varepsilon } )$ represents the tangent cone of $V_{i}$ at $\upsilon_{i}^{\varepsilon }$, and then any given $\varepsilon > 0$ is small enough. When $\upsilon^{*} + \varepsilon d \in V_{ad}$ is satisfied, we know that $\upsilon^{*}$ is the optimal control pair for (3), thus have:

17 $$\sum\limits_{i = 1}^{n} {\int_{Q} {[\left| {p_{i}^{{\upsilon_{i} + \varepsilon d_{i} }} - e_{id} } \right|^{2} + \phi \left| {\upsilon_{i}^{*} + \varepsilon d_{i} } \right|^{2} ]} } dsdt \ge \sum\limits_{i = 1}^{n} {\int_{Q} {[\left| {p_{i}^{{\upsilon_{i} }} - e_{id} } \right|^{2} + \phi \left| {\upsilon_{i}^{*} } \right|^{2} ]} } dsdt.$$

Divide both sides of inequality (17) by $\varepsilon ,$ we can know that (see, e.g.,^20,21^:

18 $$\sum\limits_{i = 1}^{n} {\int_{0}^{T} {\int_{0}^{m} {[(p_{i}^{{\upsilon_{i} + \varepsilon d_{i} }} + p_{i}^{{\upsilon_{i} }} - 2e_{id} )\frac{{p_{i}^{{\upsilon_{i} + \varepsilon d_{i} }} - p_{i}^{{\upsilon_{i}^{*} }} }}{\varepsilon } + \phi \frac{{(2\upsilon_{i}^{*} .\varepsilon d_{i} + \varepsilon^{2} d_{i}^{2} )}}{\varepsilon }]dsdt \ge 0.} } }$$

Using Lemma 1, when $\varepsilon \to 0^{ + }$, (18) can be changed to:

19 $$\sum\limits_{i = 1}^{n} {\int_{0}^{T} {\int_{0}^{m} {[(p_{i}^{{\upsilon_{i} }} - e_{id} )h_{i} (s,t) + \phi \upsilon_{i}^{*} d_{i} ]dsdt \ge 0.} } }$$

Multiply the *i*th $\left( {i = 1,2, \cdots ,n.} \right)$ equation of (15) by $h_{i}$ and integrate over Q, finally, combining (19), we conclude that:

20 $$\sum\limits_{i = 1}^{n} {\int_{0}^{T} {\int_{0}^{m} {(q_{i} (s,t) - \phi \upsilon_{i}^{*} (s,t)]d_{i} (s,t)dsdt \le 0} } } ,(\forall d_{i} \in T_{{\upsilon_{i} }} (\upsilon_{i}^{\varepsilon } )).$$

According to the definition of the normal cone, it is not difficult to obtain (see, e.g.,^22–24^:

21 $$q_{i} (s,t) - \phi \upsilon_{i}^{*} (s,t) \in T_{{\upsilon_{i} }} (\upsilon_{i}^{\varepsilon } ).$$

where, $T_{{\upsilon_{i} }} (\upsilon_{i}^{\varepsilon } )$ represents the normal cone of $\upsilon_{i}^{{}}$ in $L^{\infty } (Q)$ at $\upsilon_{i}^{\varepsilon }$, therefore, we know from the characterization of normal cone elements, it is known that the following (16) is true. In addition, in the optimal control problem (3), when $\beta_{i} (t)$ is $\beta_{i} (s,t)$, the conclusion of this paper still holds.

## Numerical simulation

The interval $Q = (0,S) \times (0,T)$ of system (1) can be divided into a grid of $N \times M$, the space step is ${S \mathord{\left/ {\vphantom {S N}} \right. \kern-0pt} N} = 0.01$ and the time step is ${T \mathord{\left/ {\vphantom {T M}} \right. \kern-0pt} M} = 0.01.$ This section uses the classic difference scheme and Newton downhill method, MATLAB program is used to solve the problem, and then the numerical simulation figures of population interaction coefficient, natural birth rate and natural death rate are drawn according to the data. The selected parameters are as follows:

$$\left\{ \begin{gathered} \lambda (s,t) = 10s^{2} (1 - s)(1 + \sin \pi t), \hfill \\ g(s) = 1 - s,m(s,t) = 0.5, \hfill \\ T = 1,l = 1. \hfill \\ \end{gathered} \right.$$

Mortality:

$$\mu (s,t) = \left\{ {\begin{array}{*{20}l} {0.06\cos^{2} (4s) + 0.03t + \frac{2 - s}{{50}}} \hfill & {0 \le s \le 2,} \hfill \\ {0.06\cos^{2} (4s) + 0.03t} \hfill & {2 < s \le 8,} \hfill \\ {0.06\cos^{2} (4s) + 0.03t + \frac{s - 8}{{10 - s}}} \hfill & {8 < s \le 10.} \hfill \\ \end{array} } \right.$$

Natural birth rate:

$$\beta (t) = \left\{ {\begin{array}{*{20}l} {0.02(\cos (t) + 1) + 0.96(2 - t),} \hfill & {1 \le t < 2,} \hfill \\ {0.02(\cos (t) + 1),} \hfill & {2 \le t < 8,} \hfill \\ {0.02(\cos (t) + 1) + 0.96(t - 8),} \hfill & {8 \le s \le 9,} \hfill \\ 0 \hfill & {Other} \hfill \\ \end{array} } \right.$$

Note in the simulation of mortality and natural birth rate to take $S = 10,T = 10$, this simulation of the effect is better.

**Figure 1:**
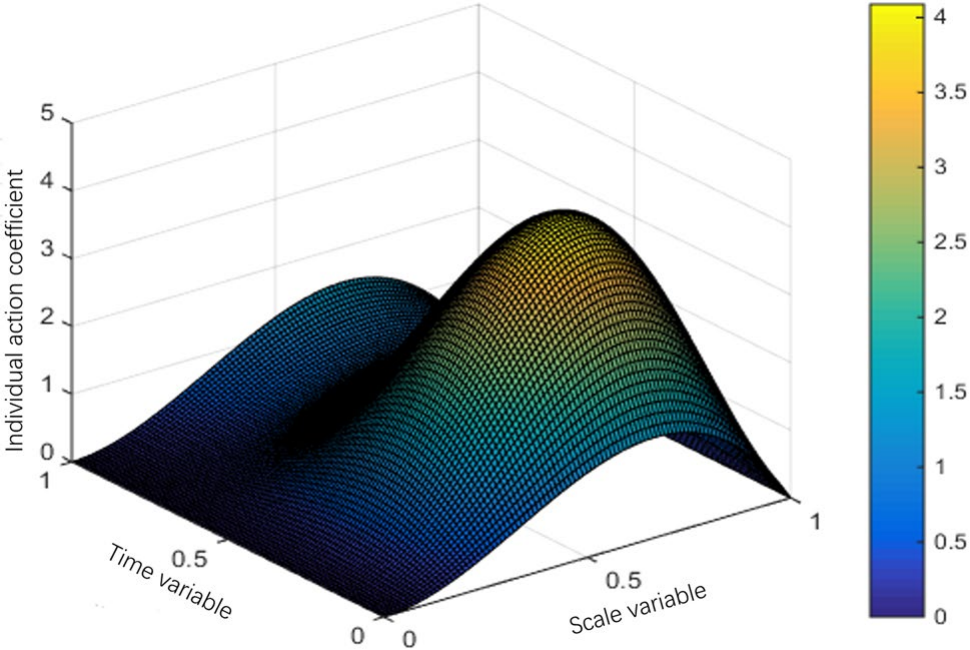
The interaction coefficients between individual scales.

**Figure 2:**
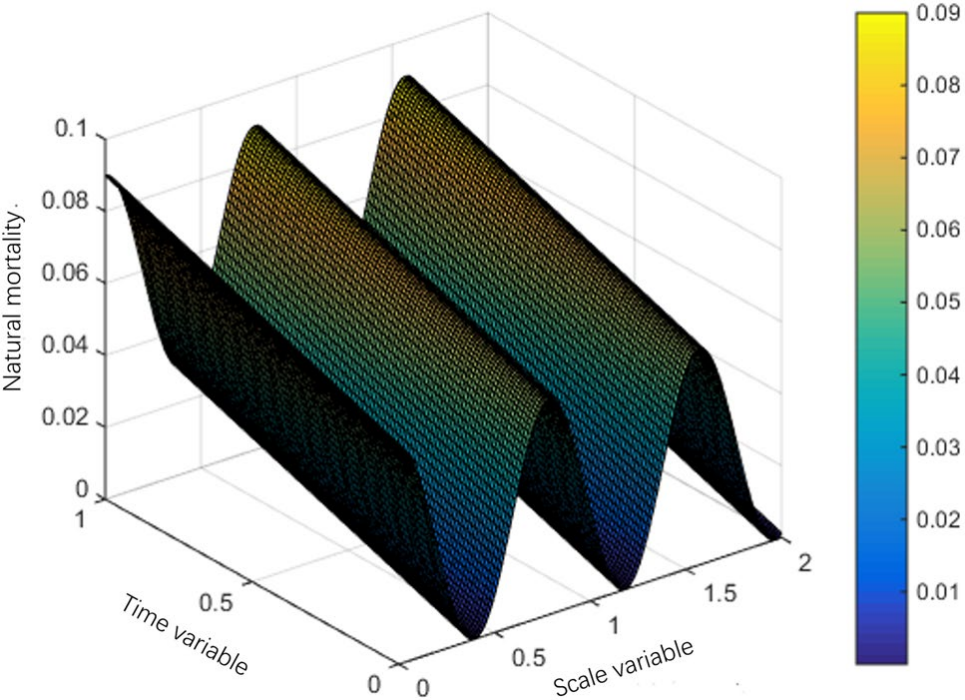
The natural mortality rate between $s \in (0,2)$.

**Figure 3:**
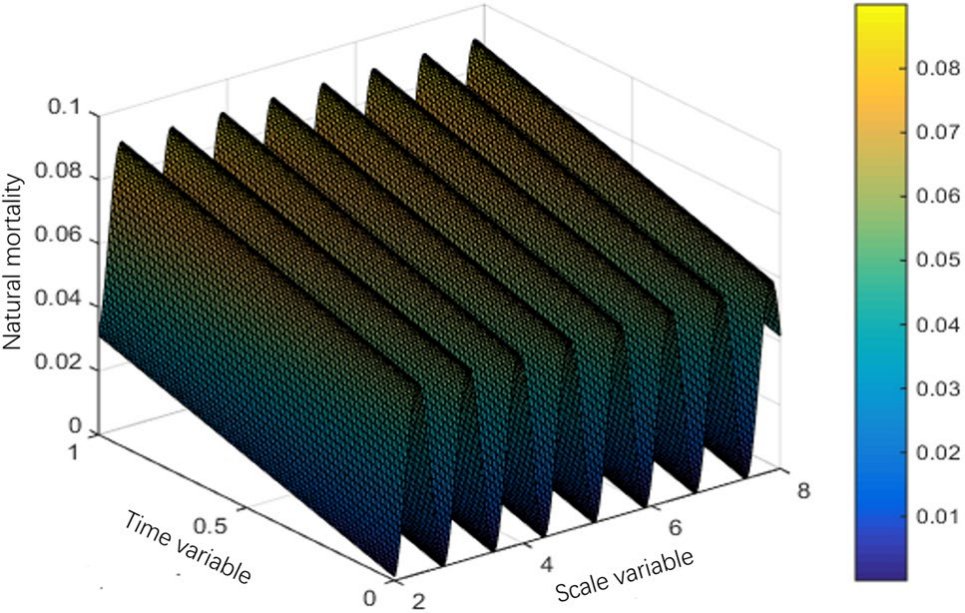
The natural mortality rate between $s \in (2,8)$.

**Figure 4:**
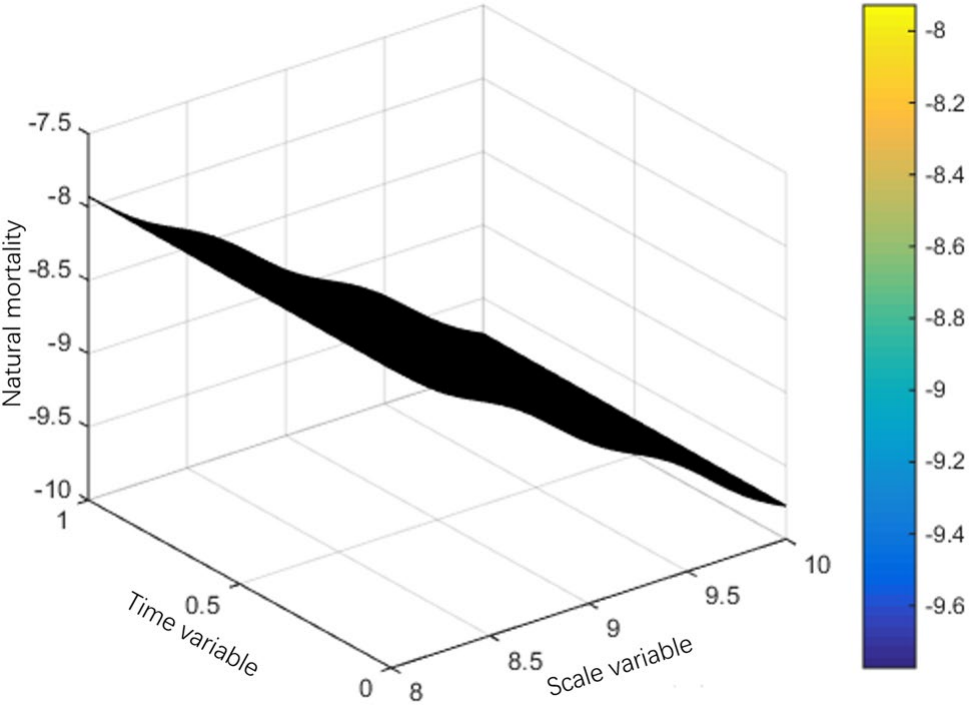
The natural mortality rate between $s \in (8,10)$.

**Figure 5:**
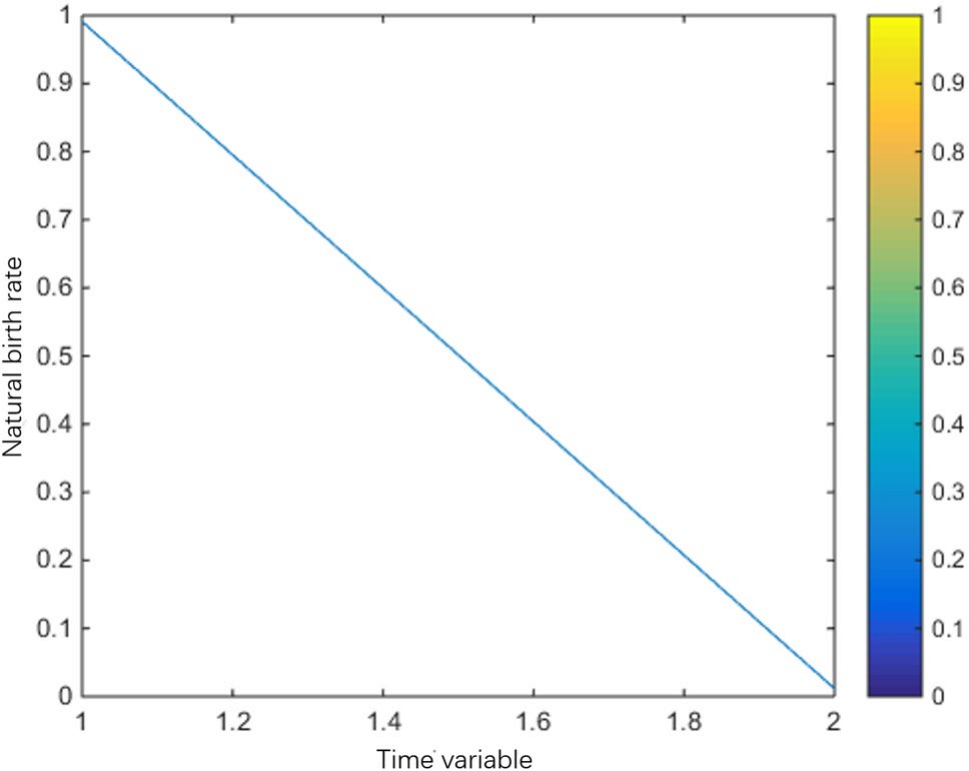
The natural birth rate between $t \in (1,2)$.

**Figure 6:**
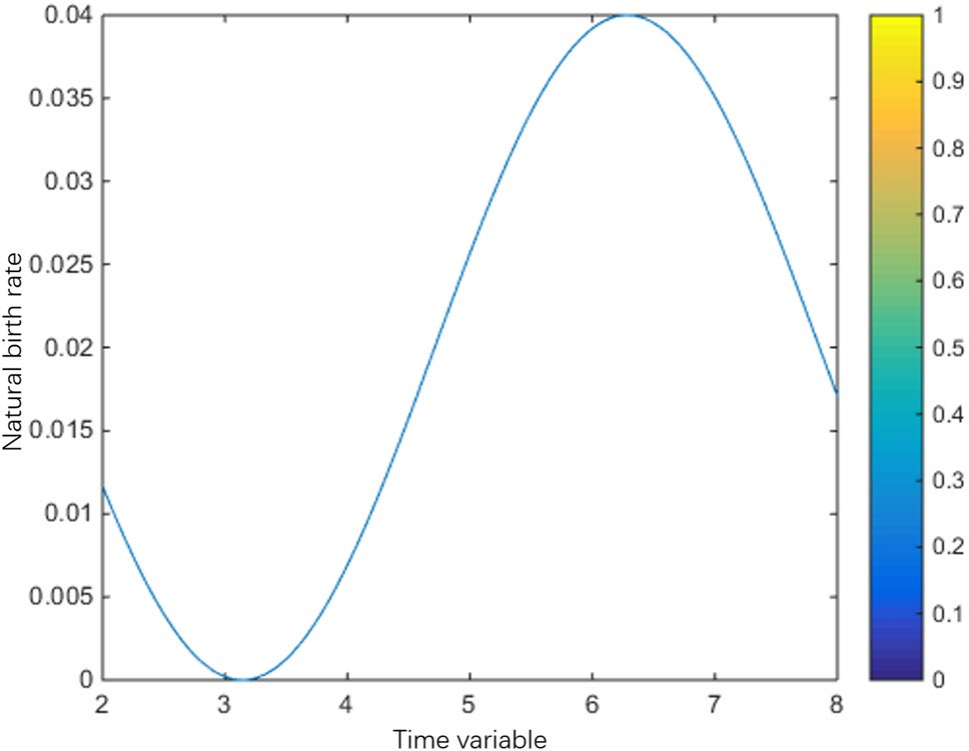
The natural birth rate between $t \in (2,8)$.

**Figure 7:**
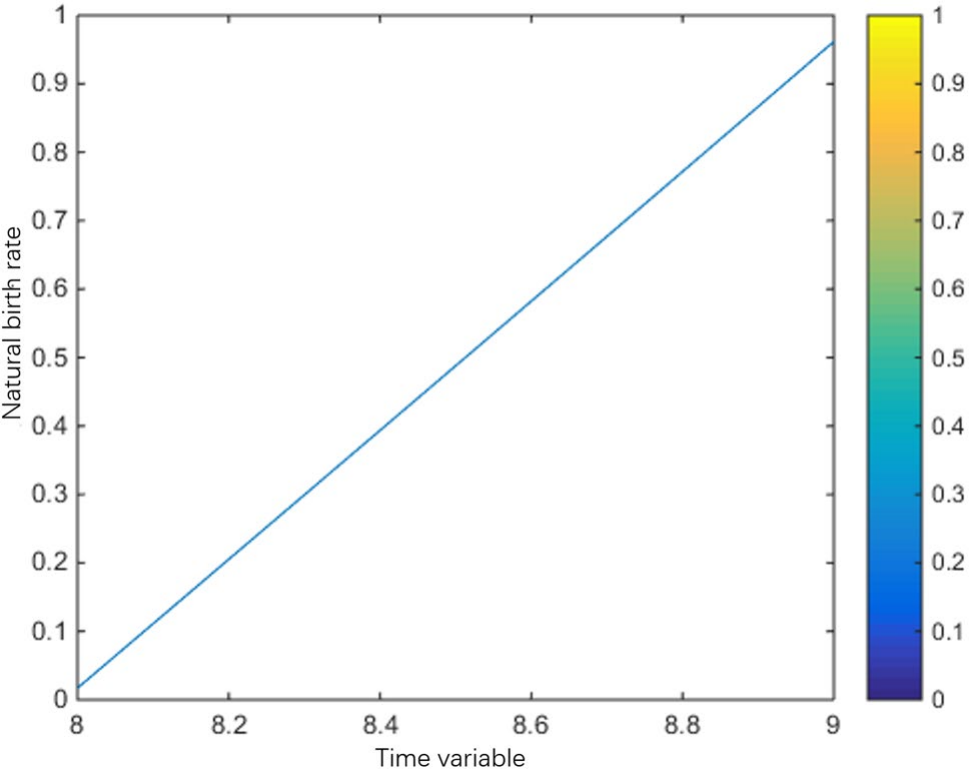
The natural birth rate between $t \in (8,9)$.

The population variation trends of interaction coefficient, reproduction rate and natural mortality rate among individuals are shown in Figs. 1, 2, 3, 4, 5, 6 and 7. When the radiance added by the environment changes to the population, the birth rate reaches the peak at the middle scale, this means that when the scale changes to its larger or smaller periods of mortality is greater, which is more consistent with the actual evolution of the population law. It can be seen that the selection of parameters $\lambda (s,t)$, $\mu (s,t)$ and $\beta (t)$ is more reasonable. In addition, which can also be seen in the numerical simulation, with the change of time,$\lambda (s,t)$, $\mu (s,t)$ and $\beta (t)$ all show the tendency of periodic fluctuation.

## Discussion

This paper is generalized on the basis of the exploitable resources of three swarm system models that depend on the individual scale distribution. Firstly, the non-negativity, boundedness, existence and uniqueness of the solution of the n-dimensional population system model relying on individual scale density distribution are discussed. Secondly, the continuous dependence on the control variables is analyzed and understood, and the existing principle of the conjugated system is deduced on the basis of the existing results. Finally, under the optimal environment of the conjugate system, with the help of the normal cone and other related knowledge, the optimality condition of the radiation control element of the n-population system model with individual scale distribution is extended. This means that it is very important to consider the radiation disturbance of the external environment to individuals, and it can provide necessary scientific theoretical analysis basis and strong practical application value for the protection of biological resources, the maintenance of ecological balance and the development, utilization and effective management of ecological resources (Supplementary Information 1).

This paper discusses the non-negativity, boundedness, existence and uniqueness of the solution of n-dimensional population system model based on individual scale density distribution; the continuous dependence of the solution of the equation on the control variables is analyzed, on the basis of the existing results, the existence principle of conjugated systems is deduced, the following conclusions can be drawn.

1. The individual scale of the population is extended to the population ecosystem model with $n(n \ge 3)$populations coexisting on the basis of the distribution of single population, two populations, and three populations in the previous literature. The optimized results show that the established mathematical model is more in line with the actual development law of natural ecological communities.
2. With the help of the normal cone and other related knowledge, the optimality condition of the radiation control element of the n-population system model with individual scale distribution is extended. This means that it is very important to consider the radiative disturbance of the individual by the external environment, and it can provide necessary scientific theoretical analysis basis and strong application value for the protection of biological resources, the maintenance of ecological balance and the development, utilization and effective management of ecological resources.
3. The optimal gestation period for the distribution of females in the population system is considered in the mathematical modeling process, On the one hand, the reproductive capacity of the population is closer to the actual biological evolution law, On the other hand, it expands the scope of application of scale structure in population systems, and provides a model basis for the study of population systems with similar scale structures.
4. The validity and feasibility of the conclusions of section "Construction of Population System Model" and section "The uniqueness of the zero-point existence of the basic food chain model function" are verified by numerical simulation In addition, according to Lemma 1, the following conclusion can be drawn: if the density distribution of $${\raise0.5ex\hbox{$\scriptstyle {q_{i} (s,t)}$} \kern-0.1em/\kern-0.15em \lower0.25ex\hbox{$\scriptstyle \phi $}} \le E_{1}$$ or $${\raise0.5ex\hbox{$\scriptstyle {q_{i} (s,t)}$} \kern-0.1em/\kern-0.15em \lower0.25ex\hbox{$\scriptstyle \phi $}} \ge E_{2}$$ population is small, it is not conducive to population reproduction and growth. Only when $$E_{1} < {\raise0.5ex\hbox{$\scriptstyle {q_{i} (s,t)}$} \kern-0.1em/\kern-0.15em \lower0.25ex\hbox{$\scriptstyle \phi $}} < E_{2}$$, the population density distribution is larger, the reproductive rate is higher, suitable for artificial scientific management.
5. If $g(s) \equiv 1,$ and $\forall (s,t) \in Q$ is satisfied, the results of this paper are in good agreement with the results of the control of the optimal radiative rate of the n-species system with age structure.

Our next research task is to apply POD order reduction algorithm and POD order reduction extrapolation method in population optimal control problem, and perform reduced-order numerical simulation error estimation analysis and graphical display of the final effect. The evolution law of the individual scale structure of organisms is determined by controlling harvest variables, finally, the benign conditions for the sustainable development of biological populations.

### Supplementary Information

Supplementary Information 1.

## Data Availability

The training data set in the manuscript is composed of random numbers (the generating method is introduced in section "Methodology" of the manuscript). The trained model we obtained will be submit as attachment (The manuscript's data.xlsx), which is also the data file needed to achieve the results of this manuscript. The other data used or analyzed during the current study available from the corresponding author on reasonable request.
